# 

**Published:** 1806-01

**Authors:** 


CONDUCTED BY

T. BRADLEY, M.D.

AND

R. BATTY, M.D.

VOL. XV.

FROM JANUARY TO JUNE, 1806.

LIBRARY
5121

*LONDON:*

PRINTED FOR RICHARD PHILLIPS, NO. 6, BRIDGE-STREET, BLACKFRIARS.

By William Thorne, Red Lion Court, Fleet Street.

W
1
ME 182

[ Entered at Stationers Hall. ]

### TO CORRESPONDENTS.

Communications are received from Dr. Patterson, Dr. Wood, and Mr. Thelwall.

